# Inflammasomes in neuroinflammation and changes in brain function: a focused review

**DOI:** 10.3389/fnins.2014.00315

**Published:** 2014-10-07

**Authors:** Gaurav Singhal, Emily J. Jaehne, Frances Corrigan, Catherine Toben, Bernhard T. Baune

**Affiliations:** ^1^Psychiatric Neuroscience Lab, Discipline of Psychiatry, School of Medicine, University of AdelaideAdelaide, SA, Australia; ^2^Discipline of Anatomy and Physiology, School of Medical Sciences, University of AdelaideAdelaide, SA, Australia

**Keywords:** inflammasomes, NLRP, neuroinflammation, cytokines, IL-1, aging, depression, Alzheimer's disease

## Abstract

Recent literature has pointed to the existence of inflammasome-mediated inflammatory pathways in central nervous system (CNS) disorders and associated changes in behavior. Neuroinflammation, which is an innate immune response in the CNS against harmful and irritable stimuli such as pathogens and metabolic toxic waste, as well as to chronic mild stress, is mediated by protein complexes known as inflammasomes. Inflammasomes activate pro-inflammatory caspases 1 and 5, which then cleave the precursor forms of pro-inflammatory cytokines IL-1β, IL-18, and IL-33 into their active forms. These pro-inflammatory cytokines have been shown to promote a variety of innate immune processes associated with infection, inflammation, and autoimmunity, and thereby play an instrumental role in the instigation of neuroinflammation during old age and subsequent occurrence of neurodegenerative diseases, cognitive impairment, and dementia. In particular, NLRP inflammasomes may also have a role in the etiologies of depression, Alzheimer's disease (AD) and in metabolic disorders, such as Type II diabetes, obesity and cardiovascular diseases that have been shown to be co-morbid with psychiatric illnesses. It has been reported that while these inflammasomes may be activated through TNF-α dependent pathways, other cytokines, like IFN-γ, may assist in inhibiting their activation and thus delay disease progression. Furthermore, some other cytokines, including IL-6, may not have a direct role in inflammasome-mediated diseases. An array of recent research suggests that NLRP inflammasomes targeted therapies could be used for alleviating neuroinflammation and for treatment of associated psychiatric illnesses, although this still remains a challenge and necessitates further extensive research. This review examines the complex inflammatory signaling pathways involved in the activation of NLRP inflammasomes and the role they play in promoting neuroinflammation and subsequent behavioral changes.

## Introduction

The discovery of inflammasomes by Martinon et al. (2002) has prompted considerable interest in the role that inflammasomes play in the mechanism of inflammation and associated disease patterns. Of late, an emerging body of literature points to the existence of inflammasome-mediated inflammatory pathways in central nervous system (CNS) disorders.

Neuroinflammation is a known factor in the pathogenesis of neurodegenerative diseases (Frank-Cannon et al., 2009), and psychiatric illnesses such as depression (Walker et al., 2014), Alzheimer's disease (AD) (Pimplikar, 2014), Parkinson's disease (PD) (Hirsch et al., 2012), Huntington's disease (Möller, 2010), and multiple sclerosis (Frohman et al., 2006). It has also been implicated in sickness behavior (Biesmans et al., 2013), diminished cognition (Ownby, 2010), and memory (Hein and O'Banion, 2009), as well as in age-related increased sensitization of the immune system to extrinsic and intrinsic stimuli (Godbout et al., 2005; Sparkman and Johnson, 2008). Pattern recognition receptors (PRRs) play an integral role in the innate immune response through recognition of pathogen specific proteins (PAMPs) and damage associated proteins (DAMPs). They are primarily expressed by glial cells, macrophages and oligodendrocytes within the brain and can be membrane bound (toll-like receptors) or within the cytoplasm [Nod-like receptors (NLRs)]. Activation of these NLRs leads to the assembly and activation of cytosolic protein complexes known as inflammasomes which then enable the activation of pro-inflammatory caspases, particularly caspase-1. This then leads to the activation of pro-inflammatory cytokines interleukin (IL)-1β, IL-18, and IL-33 (Arend et al., 2008; Chakraborty et al., 2010), which promote a number of innate immune processes associated with infection, inflammation and autoimmunity (Davis et al., 2011), thereby responsible for neuroinflammation and associated brain diseases (Tha et al., 2000; Cacquevel et al., 2004; Felderhoff-Mueser et al., 2005; Godbout and Johnson, 2009; Mawhinney et al., 2011; Zhang et al., 2014).

It has been known for some time that immunosenescence, in addition to neurodegenerative changes with age, predisposes the brain to higher risk of acquiring neuroinflammatory disorders. Considerable findings during the last decade have suggested an instrumental role of inflammasomes in the pathophysiology of neuroinflammation during neuronal ageing, and its associated neurodegenerative diseases such as dementia, leading to loss of memory and cognitive impairment (Simi et al., 2007; Chakraborty et al., 2010; Mawhinney et al., 2011; Liu and Chan, 2014). In particular, NLRP (NLR family, containing pyrin domain) inflammasomes have been shown to have a role in the etiologies of several neurological diseases such as depression (Zhang et al., 2014), AD (Tan et al., 2013), PD (Cedillos, 2013), and multiple sclerosis (Gris et al., 2010; Fischer et al., 2012). Systemically, NLRP inflammasome-driven inflammatory responses also play a role in the development of Type II diabetes (Grant and Dixit, 2013; Lee et al., 2013), obesity (Stienstra et al., 2011), and cardiovascular diseases (Garg, 2011), as well as cancer (Zitvogel et al., 2012). Given that metabolic disorders can predispose to the development of psychiatric disorders, it is possible that inflammasome-driven inflammatory pathways may be a potential mechanism driving this co-morbidity.

A number of studies have investigated the innate immune pathways associated with the activation of NLRP inflammasomes and the subsequent production of IL-1β, IL-18, and IL-33 from their precursors. The aim of this review is to examine these complex inflammatory signaling pathways associated with NLRP inflammasomes activation, and leading to neuroinflammation and behavioral changes that have commonly been observed during various psychiatric disorders and brain aging.

## Materials and methods

### PRISMA criteria

The guidelines prescribed by PRISMA (Preferred reporting items for systematic reviews and meta-analyses) (Liberati et al., 2009; Moher et al., 2009) were followed while constructing this review. The checklist items from PRISMA as relevant to this review, for example those related to search and writing approaches, were included and the items not relevant, for example those related to meta-analyses, were excluded.

### Search and selection process

**Figure 1:**
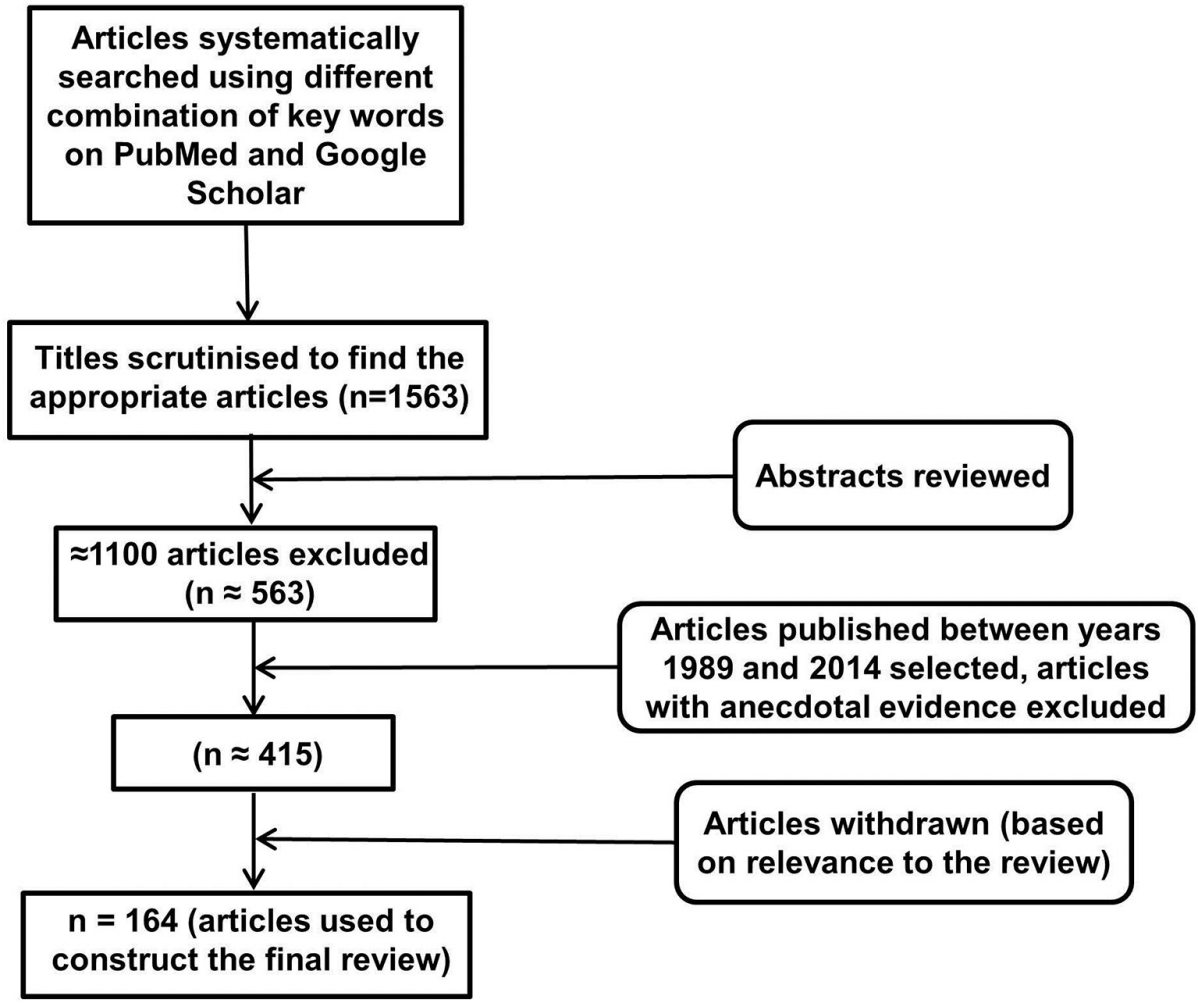
**Study inclusion flowchart**. It depicts the methodology for search and collection of relevant articles for this review, following PRISMA guidelines (Liberati et al., 2009; Moher et al., 2009).

An electronic database search of PubMed and Google Scholar with several key terms in various permutations was performed. These included but were not limited to: inflammasomes, neuroinflammation, NLRP, NALP (NACHT, LRR, and PYD domains containing proteins), cytokines, IL-1, IL-18, IL-33, TNF, cellular, humoral, immune, aging, depression, AD, PD, Huntington's disease, multiple sclerosis, cognition, behavior, metabolic disorders, diabetes, obesity, cardiovascular disease, cancer, pathogen associated molecular patterns, damage associated molecular patterns, toll like receptors, and glial cells. At each stage of the search, titles and abstracts were scrutinized and the most appropriate organized into separate folders using End Note X6.0.1 software. In addition, articles relevant to our discussion were retrieved from the reference list of other online articles on each subtopic. This in total yielded 1563 papers. After placing all inclusion and exclusion criteria into our search (depicted in Figure 1), 164 articles closely related to the aims set forth for this review were selected and hence utilized.

### Inclusion and exclusion criteria

The emphasis of this review has been on inflammatory pathways associated with inflammasome activity in the brain, and as such articles investigating inflammasomes, in particular NLRP inflammasomes and their mechanism of actions in CNS disorders were selected for detailed analysis. In addition, articles addressing the effects of IL-1 family of cytokines in the brain were read thoroughly to understand and analyze the various mechanisms of actions of these cytokines, especially in the brain and their association with inflammasomes. Other immune factors related to inflammasomes and the role of inflammasomes in systemic diseases was also investigated while writing this review. All articles included in this review have been published between 1989 and 2014. Articles without the full text available and with anecdotal evidence were excluded from the review.

## Structure of NLRP inflammasomes

The NLR family, pyrin domain containing inflammasomes (NLRP) are the most studied and best characterized protein complex during inflammation (Stutz et al., 2009). NLRs are intracellular PRRs and function in association with Toll-Like Receptors to sense the presence of PAMPs which are found in a variety of microorganisms that enter cell through phagocytosis (infectious stimuli), and DAMPs such as nuclear and cytosolic protein characteristics of tissue injury/stress (non-infectious stimuli). In turn, this activates the innate and acquired immune response (Inohara et al., 2005; Kanneganti et al., 2007; Franchi et al., 2009). A key part of this process is the assembly of inflammasome complexes that generally have three main components: a cytosolic PRR (either from the NLR family or the pyrin and HIN domain containing family-PYHIN), caspase-1 and an adaptor protein ASC (apoptosis-associated speck like protein). The NLR family contains a leucine rich repeat domain (LRR), a central NACHT domain and a variable amino-terminal domain, which in the NLRP subfamily is an N-terminal pyrin domain (PYD). Activation of NLRPs leads to the recruitment of ASC which contains a caspase activation and recruitment domain (CARD). ASC then interacts with the CARD of pro-caspase-1. There are exceptions to this sequence, with for example NLRP1, directly interacting with pro-caspase 1, without necessarily needing ASC. Nonetheless the interaction with pro-capse-1 leads to its conversion to caspase 1, which then converts pro forms of IL-1β, IL-18, and IL-33 into their active forms, initiating an inflammatory response (Martinon et al., 2002; Petrilli et al., 2005). The NLRP3 is the largest and most studied inflammasome of all known at this stage (Stutz et al., 2009).

**Figure 2:**
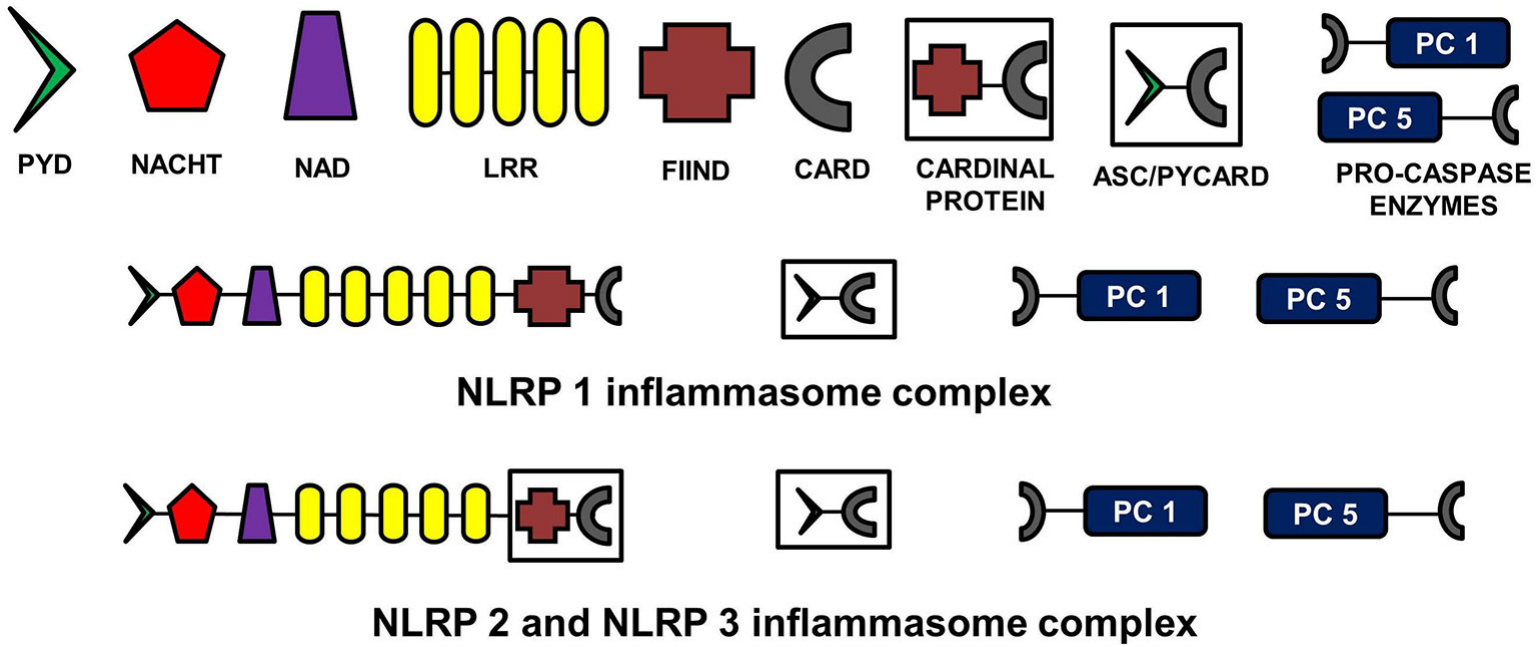
**Structure of NLRP inflammasomes**. NLRP inflammasomes are intracellular protein complexes consisting of NLRP (NACHT, LRR, and PYD domains containing proteins 1, 2, or 3), the adapter protein ASC/Pycard, enzyme pro-caspases 1 and 5, and cardinal proteins. NLRP1 has Pyrin (PYD) domain on the amino (N)-terminal. This PYD domain is bonded to a NACHT domain followed by a NACHT-associated domain (NAD), several lucine-rich repeats (LRR), FIIND domain and the caspase recruitment domain (CARD) at the carboxy (C)-terminal. The molecular structures of NLRP2 and NLRP3 are similar to NLRP1, except that instead of being directly linked to FIIND domain, LRRs are linked to a cardinal protein which consists of FIIND domain on N-terminal and CARD domain on the C-terminal. NLRPs with the adapter protein ASC/Pycard and pro-caspase enzymes form the inflammasome complex responsible for converting pro-IL-1 cytokines into their active forms within the cytoplasm of glial cells primarily. ASC, apoptosis-associated speck-like protein containing a CARD.

See Figure 2 for the schematic representation of the structures of different NLRP inflammasomes as described above.

## The role of IL-1 family of cytokines in inflammation, pathological states, and homeostatic response in the brain

Pro-inflammatory cytokines function to attract leucocytes and enhance their proliferation at the site of inflammation. They stimulate cytotoxicity, release of proteolytic enzymes, synthesis of prostaglandins, and synthesis and secretion of secondary cytokines. This in turn promotes inflammation and increases thermoregulatory set point, generally associated with symptoms such as fever, tissue destruction, shock, and even death (Cannon, 2000). The IL-1 family of cytokines comprises 11 secreted factors, including IL-1α, IL-1β, IL-18, and IL-33, which are known for playing a role in host defense and immune system regulation in inflammatory diseases (Barksby et al., 2007; Arend et al., 2008; Dinarello, 2009; Sims and Smith, 2010). These cytokines have been shown to be involved in a variety of immune reactions as well as in the initiation, regulation, and maintenance of inflammation (Dinarello, 2000). In particular, cytokine mediated processes have been shown to result in long term neuropsychiatric disorders and were found to be related to major depression, dementia, and AD (Licastro et al., 2000; Cacquevel et al., 2004; McAfoose and Baune, 2009). The presence of IL-1β has been demonstrated in cerebrospinal fluid and plasma of patients with AD (Licastro et al., 2000; Tarkowski et al., 2003). Similarly, the roles of IL-18 and IL-33 in neuroinflammation and neurodegenerative diseases have also been well established (Felderhoff-Mueser et al., 2005; Arend et al., 2008; Liew et al., 2010).

However, it is important to note that although pro-inflammatory cytokines (IL-1 and TNF family of cytokines) have been shown to result in neuroinflammation and neurodegenerative diseases when expression is high, at constitutive levels they are required for normal physiological functioning, particularly in the molecular and cellular mechanisms responsible for learning, memory and cognition (McAfoose and Baune, 2009). They influence and maintain homeostasis in monoamine metabolism, neuronal genesis and survival, Hypothalamic-Pituitary-Adrenal (HPA) axis sensitivity to cortisol and certain cellular neuroimmune functions (Eyre and Baune, 2012). However, levels of both pro-inflammatory and anti-inflammatory cytokines have been shown to be elevated in many brain disorders, including AD, PD, and age related dementia, indicating their role in cognitive and memory deficits with age. When pro-inflammatory cytokines are overexpressed, anti-inflammatory cytokines potentially function to suppress the gene expression for pro-inflammatory cytokine production and control the pro-inflammatory response. For instance, gene knockout mice for anti-inflammatory cytokines, such as IL-1ra, IL-10, and TGF-β 1 showed enhanced inflammatory reactions (Dinarello, 2000). However, no study describes the effects of anti-inflammatory cytokines on activated inflammasomes. Activated microglia and astrocytes are the main source of cytokines in the brain (Rothwell et al., 1996; Hanisch, 2002).

**Figure 3:**
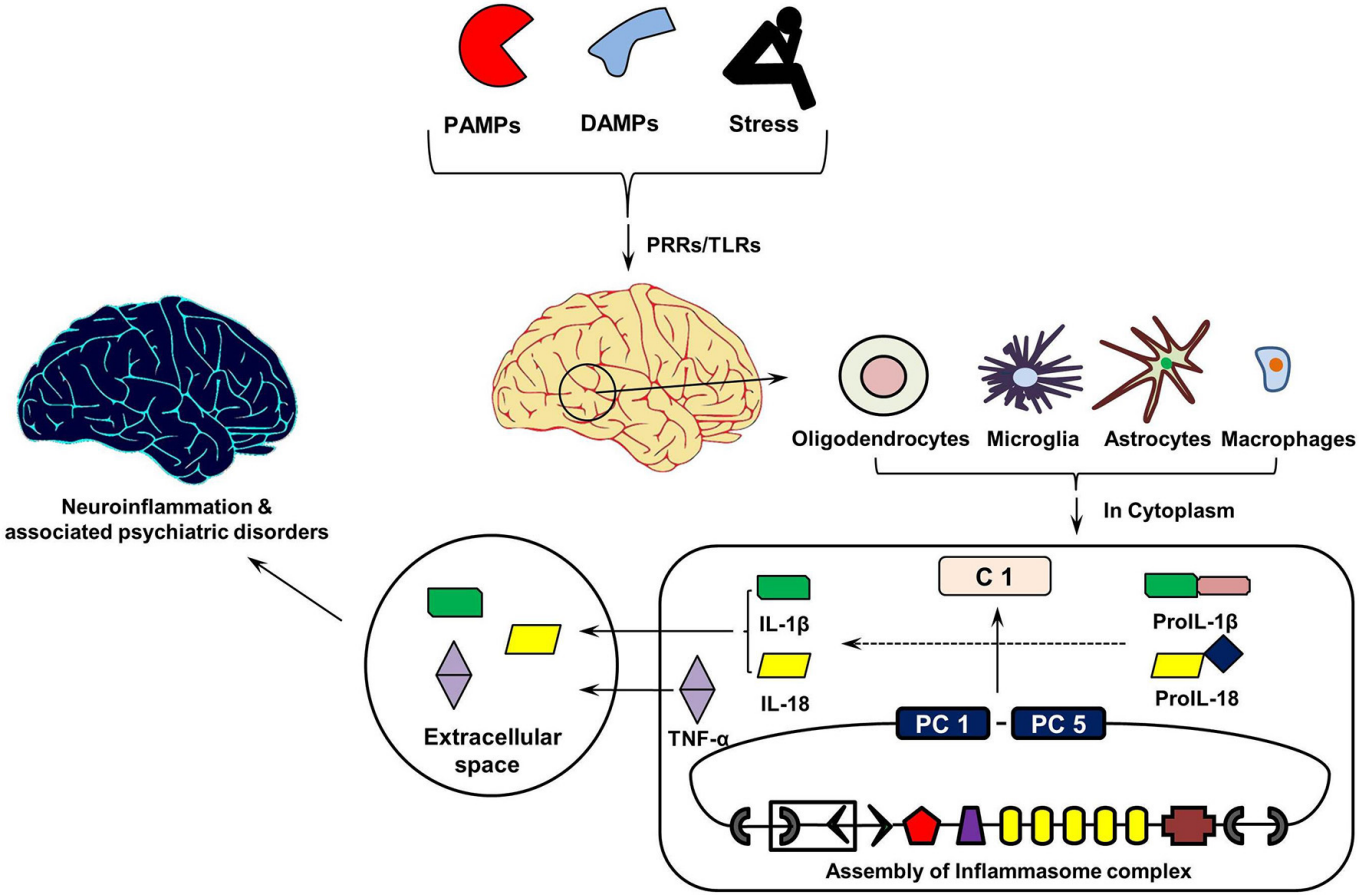
**Inflammasomes Cascade in Brain**. On recognizing pathogen associated molecular patterns (PAMPs, found in phagocytized microorganisms) and damage associated molecular patters (DAMPs, e.g., nuclear and cytosolic proteins), intracellular pathogen recognition receptors (PRRs) and Toll-Like Receptors (TLRs) initiates assembly of cytosolic inflammasome complex. In turn, this activates the innate and acquired immune responses involving Interleukin (IL)-1 cytokines, which in addition to Tumor Necrosis Factor (TNF)-α initiate inflammatory reaction in the extracellular space. PC1 and PC5, Pro-caspases 1 and 5; C1, Caspase 1.

Figure 3 shows the cytokine cascade in brain following stimulation with an infectious agent, metabolic waste or foreign material. As this includes increase in the levels of IL-1β cytokine which can only be activated from its precursor in the presence of enzyme caspase 1, it indicates an active involvement of inflammasome action during this cytokine cascade.

### Link between aging of brain, IL-1 cytokines, and brain disorders

Aging of the brain has been shown to be associated with many cognitive and memory deficit disorders and is believed to be regulated by extrinsic (e.g., environmental) and intrinsic (e.g., genotype) factors (van der Staay, 2002). Several brain disorders, such as AD and PD, are the products of chronic neuroinflammation and resultant neurodegeneration (Heneka et al., 2010; Hirsch et al., 2012), the symptoms which are also common to the aging brain (McGeer and McGeer, 2004). Dementia, decline in cognitive abilities and impairment of spatial memory are often seen during aging and are associated with neuroinflammatory changes within the brain accumulated over a period of time. Indeed, a significant association has been found between age related depression and level of pro-inflammatory cytokines in the brain (Godbout et al., 2008). Moreover, the risk of infections increases with age, mainly due to immunosenescence (Aw et al., 2007) and a rise in circulating autoantibodies and lymphoproliferative disorders, hence contributing toward greater morbidity and mortality in old age (Shinkai et al., 1998; Senchina and Kohut, 2007).

Structural and functional changes in the brain are coordinated by a range of intracellular signaling molecules. Consistent findings suggest that an increase in the expression of pro-inflammatory cytokines by astrocytes and microglia within the brain results in neuroinflammation followed by neurodegeneration, eventually resulting in cognitive and memory deficit and exacerbated sickness and depressive-like behavior (Mrak and Griffin, 2005; Huang et al., 2008). Microglia are primed with aging and upon secondary stimulation, these microglia release excessive quantities of pro-inflammatory cytokines, such as TNF-α, IL-1β, and IL-6 (Dilger and Johnson, 2008). The increase in the number of microglia, astrocytes and percentage of GFAP in the brain with age has been reported in rodents, which is subsequently found to be related to cognitive and memory impairment (Sugaya et al., 1996; Rozovsky et al., 1998), and to neurodegenerative diseases such as AD (Mrak and Griffin, 2005). The effects of aging on microglial functions in the brain has been reviewed in detail by Conde and Streit (2006). During aging, glial cells, particularly microglia show increased activation and expression of pro-inflammatory cytokines, such as IL-1, however, they also become increasingly dysfunctional and lose neuro-protective properties which predispose the brain to the neurodegenerative disorders in line with other genetic and acquired environmental risks (Mrak and Griffin, 2005; Streit, 2005). Similarly, astrocytes are the immune effector cells, which express cytokines (IL-1, IL-6, IL-10, interferons α and β, TNF α and β) and chemokines, and mediate inflammation and immune reactivity in the brain. The under-expression or over-expression of astrocytes can also lead to neurodegenerative diseases (Dong and Benveniste, 2001).

### Inflammasome mediated inflammatory pathways in the aging brain

It was not known until recently whether inflammasomes play any role in causing or aggravating neuroinflammation during neuronal aging. Considerable recent findings have suggested an instrumental role of inflammasomes in the pathophysiology of neuroinflammation during neuronal ageing, and associated neurodegenerative diseases, cognitive impairment and dementia (Simi et al., 2007; Chakraborty et al., 2010; Mawhinney et al., 2011; Liu and Chan, 2014). There is upregulation in the expression of several genes that signal inflammasome assembly and activation of caspase 1 (e.g., thioredoxin-interacting protein, P2X7, and pannexins), as well as signaling of TLRs (e.g., CD14, TLR2, TLR4, TLR7, TOLLIP, MYD88) in different regions of the brain such as hippocampus, post-central gyrus, and superior frontal gyrus during aging (Cribbs et al., 2012). TLRs are evolutionary conserved microbe specific structural motifs (e.g., PAMPs) and endogenous molecule (e.g., DAMPs) recognition transmembrane or endosomic membrane proteins, primarily expressed in various sentinel cells such as dendritic cells, macrophages, and plasmatoid dendritic cells that form the first line of defense (Kumar et al., 2009). The interaction between TLRs and LRRs on cytosolic NLRs trigger the assembly and activation of inflammasomes culminating in caspase 1 catalyzing pro-IL-1 cytokines into their active forms (van de Veerdonk and Netea, 2011). The increased activity of both TLRs and NLRs in the aging brain can therefore act as a deterrent to the negative regulation of their expression and a sustained expression could result in chronic neuroinflammation and associated neurodegenerative diseases. Moreover, production of reactive oxygen species (ROS) from dysfunctional mitochondria and increased NF-κB signaling with aging could also potentiate the priming of NLRP3 inflammasomes in the brain resulting in an inflammatory response (Salminen et al., 2012). Formation of mutant α-synuclein and Aβ fibrils, seen during PD and AD respectively, further pose greater danger in old age as they act to enhance the activity of inflammasomes in the brain (Salminen et al., 2009; Tschopp and Schroder, 2010; Cedillos, 2013).

Enhanced inflammasome activity could manifest in the form of cognitive decline in an aging population, as shown in 18 month old male Fisher 344 rats, however spatial learning improved when rats were treated with an anti-inflammatory drug probenecid (Mawhinney et al., 2011) which reduced NLRP1 inflammasome activation. However the mechanism for this is not yet completely understood.

## Association between neuroinflammation and major depressive disorder

Major depressive disorder (MDD) is characterized by a distinct change of mood accompanied by sadness, irritability, loss of interest in all activities and events, as well as psychophysiological changes (Belmaker and Agam, 2008). A number of studies, both experimental and meta-analytic, have revealed increased expression of pro-inflammatory cytokines, TNF-α, IL-1β, and IL-6, in the brain of MDD patients leading to neuroinflammation (Maes et al., 1997; Howren et al., 2009; Dowlati et al., 2010; Hannestad et al., 2011). Role of chronic mild stress in neuroinflammation and subsequent occurrence of depression has also been established (Farooq et al., 2012), although it is not clear if inflammasomes have any role in causing neuroinflammation in response to chronic mild stress.

### Inflammasome mediated inflammatory pathways associated with major depressive disorder

The increase in IL-1β levels and neuroinflammation in the brain of MDD patients potentially suggests a role for inflammasomes in MDD. Researchers have indeed recently shown the involvement of the NLRP3 inflammasome in lipopolysaccharide (LPS)-induced mouse depressive-like behavior (Zhang et al., 2014). Similar findings were seen in human participants when activated NLRP3 inflammasomes were detected in blood mononuclear cells from depressive patients (Alcocer-Gómez et al., 2014). To investigate the etiological role of inflammasomes in depression, a panel of researchers conducted a clinical trial in major depression and schizophrenic patients. They concluded from the study that inflammasome-related inflammation is an ongoing process in psychiatric patients during diseased states (Hohmann et al., 2014). Moreover, the recent finding that mice lacking caspase-1 are resistant to LPS-induced depressive-like behavior further supports the role of inflammasomes in depression (Moon et al., 2009). Some authors recently reviewed the role of inflammasomes in MDD and its comorbidity with systemic illnesses, and proposed a new inflammasome hypothesis of depression and related comorbid systemic illnesses (Iwata et al., 2013). The review highlights the central mediator role that inflammasomes play in the contribution of psychological and physical stressors to the development of depression and its association with systemic illnesses. The activation of inflammasomes, particularly NLRP3, therefore could be indirectly related to the pathophysiology of depression and its comorbidity with other systemic diseases through an inflammatory response in the brain.

## Association between neuroinflammation and Alzheimer's disease

AD is characterized by a debilitating chronic and progressive neurodegeneration leading to major clinical hallmarks of loss of memory, cognitive deficit, dementia, and behavioral impairment. Although, the prevalence of AD is higher in people over 60 and it increases proportionally with every 10 years of age (Younger/Early Onset Alzheimer's and Dementia: Alzheimer's Association, 2014), early onset of AD has also been reported in people in their 40s and 50s (Kim et al., 2014). Several factors such as genetic predisposition (Bertram and Tanzi, 2009; Kamboh et al., 2012), reduced synthesis of excitatory neurotransmitter acetyl choline (Babic, 1999), extracellular deposition of amyloid beta (Aβ) in the brain (Palop and Mucke, 2010), abnormalities in tau protein forming neurofibrillary tangles leading to disintegration of microtubules (Ballatore et al., 2007), and oxidative stress and inflammatory cascades mediated by primed glia cells (Agostinho et al., 2010) have been proposed to cause AD. These different hypotheses have been established after years of independent research; however, recent efforts toward finding the common link between the causal factors for AD have pointed toward the inflammatory cascade linking them in the brain. Indeed, damaged neurons, highly insoluble Aβ deposits and neurofibrillary tangles could provide stimuli for neuroinflammation (Wenk, 2003). Similarly, neurotransmitter acetylcholine has been shown to be involved in inhibiting the release of pro-inflammatory cytokines from microglia and monocytes (Tabet, 2006), an anti-inflammatory mechanism that could be disturbed during acetylcholine deficiency. This suggests that all above etiologies for AD, when accompanied by chronic neuroinflammation, lead to progressive neurodegeneration and behavioral impairment with age, characteristics of symptoms of AD. In the absence of neuroinflammation, these etiologies may not provide sufficient pathology to cause AD. This is supported by the finding that significant amyloid deposition could be present in the brain of healthy elderly individuals without cognitive impairment (Aizenstein et al., 2008). Likewise, while higher quantities of tau proteins have been reported in the brain of AD patients than unaffected individuals (Avila et al., 2004), some authors have challenged the tau protein hypothesis and proposed that tau phosphorylation is a compensatory mechanism to protect neurons against oxidative stress (Lee et al., 2005). Nonetheless, this suggests overall that a single factor alone may not be sufficient to cause AD, and irrespective of the causative factor, neuroinflammation essentially provides a central pathway to the onset of AD which is mediated by various pro-inflammatory cytokines and chemokines, including IL-1 family of cytokines that are activated by inflammasomes.

Indeed, IL-1β and IL-18 over-expression has been shown to initiate inflammatory process in the brain of AD patients (Rubio-Perez and Morillas-Ruiz, 2012; Liu and Chan, 2014). This over-expression has been detected in microglia, astrocytes as well as neurons, and found to be co-localized with both Aβ plaques and tau. Interestingly, it has also been suggested that chronic inflammation could be the cause for increase in Aβ and tau phosphorylation in the brain (Meraz-Ríos et al., 2013). In support of this, studies on transgenic mice with LPS-induced neuroinflammation have shown enhanced intracellular deposition of Aβ (Sheng et al., 2003; Lee et al., 2008) and tau phosphorylation (Kitazawa et al., 2005) in the brain of mice. Overall, this suggests a chain of continuous adverse events in the brain of AD patients, mediated by IL-1 family of pro-inflammatory cytokines.

### Inflammasome mediated inflammatory pathways associated with Alzheimer's disease

Recently, the role of inflammasomes, particularly NLRP3, in oxidative stress-induced neuroinflammation and impaired amyloid metabolism seen in AD brains has been evaluated and recognized (Halle et al., 2008; Marchesi, 2011; Tan et al., 2013). Neuronal injury caused by insoluble Aβ oligomers and fibrils releases DAMPs which are sensed by PRRs (NLR domain) on NALP inflammasomes initiating a chain of events leading to the maturation of proIL-1β and proIL-18 cytokines and release of their active forms as the final event (Halle et al., 2008; Salminen et al., 2009). Moreover, Aβ can interact with neuronal membranes to create ion channels that allow potassium ion (K^+^) efflux mediated by ATPase enzyme, activating inflammasomes and in turn secretion of the active IL-1 family of cytokines (Salminen et al., 2009; Tschopp and Schroder, 2010). Reduction in intracellular K^+^ to 90 mM though has been found to be a requirement for the activation of NLRP3 inflammasomes (higher intracellular concentration of K^+^ inhibits activation of inflammasomes) (Petrilli et al., 2007). Some authors however, found impaired activity of Na^+^/K^+^ ATPase in AD patients (Hattori et al., 1998) that is required for the active efflux of K^+^ across the cell membranes, which therefore raises the question whether efflux of K^+^ is essential for the activation of inflammasomes. ATPase is required to catalyze ATP into ADP and a phosphate ion with the release of energy that activates the purinergic P2X_7_ receptor. This receptor in turn decreases intracellular K^+^ levels (Perregaux and Gabel, 1994; Solle et al., 2001). Purinergic signaling has also been shown to control the cerebral vascular tone and this has been implicated in learning and memory, locomotor and feeding behavior and sleep (Burnstock, 2013). Nonetheless, diminished activity of Na^+^/K^+^ ATPase reduces the gradient of ions across the cell membranes causing an excitotoxic cellular response resulting in neuronal death (Hattori et al., 1998). This causes a release of DAMPs from dead neurons that potentially act as the activators of NLRP3 inflammasome dependent innate immune response (Rubartelli, 2014). It has also been shown that disease-associated extracellular amyloid and unique protein aggregates caused by inappropriate oligomerization or misfolding are sensed by NLRP3 inflammasomes (Masters and O'Neill, 2011), likely as DAMPs within the resident macrophages after engulfment in the brain. Research has also suggested that the brain in AD is under increased oxidative stress and Aβ peptides generates free radicals that together further enhance neuron degeneration and death (Markesbery, 1997). Mitochondrial ROS released during tissue injury/death could enhance oxidative damage and signal inflammasome activation up-regulating pro-inflammatory cytokine levels in brain (Martinon, 2010; Tschopp and Schroder, 2010; Naik and Dixit, 2011), potentially resulting in neuroinflammation.

Significant pathology and behavioral deficits characteristics of chronic neuroinflammation do not manifest until advanced age. This has been attributed to the capacity of the brain to compensate for the presence of chronic neuroinflammation by regulating the glutamatergic system (Brothers et al., 2013). This suggests that neuroinflammation in itself does not cause AD; however it acts as an initiator, enhancer and sustainer of AD disease during old age which is reinforced by various other etiologies. Since IL-1 cytokines are key contributors of chronic neuroinflammation and associated neurodegenerative diseases, including AD, inflammasomes provide a possible answer for the mechanism of IL-1 action and the ways in which IL-1 activity is regulated during chronic neuroinflammation in old age (Allan et al., 2005).

## Immune factors associated with the activity of inflammasomes

It has been well established that both TNF-α and IL-1β stimulate each other's secretion and exhibit overlapping and synergistic effects (Akira et al., 1990; Ikejima et al., 1990; Knofler et al., 1997). For instance, while TNF-α enhances migration of leucocytes in inflamed tissue and promotes apoptosis, IL-1β acts as a potent pyrogen and decreases the threshold of pain by inducing the transcription of cyclooxygenase 2 enzyme, thereby enhancing production of prostaglandins E_2_, which is responsible for pain and fever. This raises a possibility that inflammasomes, which catalyze IL-1β precursors, may stimulate TNF-α secretion through an indirect pathway. Contrary to this, it has recently been reported that inflammasomes may also be activated independently of PRRs, through TNF-α dependent pathways. TNF-α has been shown to trigger the activation of caspase 1 and in turn secretion of IL-1β (Alvarez and Munoz-Fernandez, 2013), suggesting a possible bidirectional cause-effect relationship between inflammasomes and TNF-α. Although the precise mechanism for this has not yet been elucidated, this study indicated that TNF-α may potentially substitute for a TLR mediated stimulus required for inflammasome activation. Moreover, recent findings also suggest that TNF-α induces production of IL-33 in keratinocytes (Taniguchi et al., 2013) and regulates expression of IL-18 in dendritic precursor-like cell line KG-1 and cardiomyocytes (Chandrasekar et al., 2003; Koutoulaki et al., 2010), further supporting the hypothesis that TNF-α has a role in inflammasome activation, although this direct relation between TNF-α, and IL-18 and IL-33 is yet to be established in the brain. Furthermore, other cytokines such as Type I interferon (IFN) gamma have been shown to inhibit caspase-1 cleavage and reduce IL-1β secretion in rodents (Guarda et al., 2011). Conversely, although IFN-gamma does not cause inflammation by activating inflammasomes directly, it has been shown to augment TNF activity (Dinarello, 2000). Although IL6 is a reliable inflammatory marker it may not always be directly involved in inflammasome mediated inflammation as seen in an IL-6 knock in mouse model (McGeough et al., 2012). Overall, this suggests a complex and intricate immune pathway mediated by various cytokines that may be involved in the activation of inflammasomes in the brain, and resultant neuroinflammation and changes in brain function; however this requires validation through extensive research.

## Inflammasome-independent neuroinflammation-mediated brain pathologies

TNF-α is another pro-inflammatory cytokine, in addition to IL-1 cytokines, that has been primarily implicated in neuroinflammation. Elevated levels of TNF-α in particular have been shown to cause a reduction in hippocampal volumes through the neurodegenerative TNFR1 pathway (Baune et al., 2012) and can lead to the development of depressive-like behavior (Eyre et al., 2013). Glial cells, microglia, and astrocytes, are the primary immune effector cells and express various cytokines in the CNS (Rothwell et al., 1996; Hanisch, 2002). Though glial cells are neuroprotective, their over expression or sustained stimulation can result in enhanced production of cytokines (e.g., IL-1β and TNF-α) (Sawada et al., 1989; Dong and Benveniste, 2001; Hanisch, 2002) resulting in severe neuroinflammation, neurodegeneration and subsequent cognitive dysfunction and psychiatric diseases, such as AD.

While levels of both pro-inflammatory and anti-inflammatory cytokines in the peripheral circulation and CNS have been reported to rise during several brain disorders such as depression, schizophrenia and AD (Schwarz et al., 2001), other humoral immune factors, such as mitogen-activated protein kinases (MAPK), C reactive protein (CRP), the complement system and chemokines have also been reported to modify brain anatomy and functions. MAPKs are specific protein kinases (serine-threonine specific) that elicit pro-inflammatory and immunomodulatory functions (Lee et al., 1994; Dong et al., 2002). Similarly, CRP is an acute phase reactant protein which enhances inflammation and tissue damage by promoting phagocytosis by opsonization (Du Clos, 2000) and activating the complement system (Padilla and Perez, 2003). High levels of CRP in the brain have been linked to neuroinflammation and associated cognitive impairment and dementia (Kuo et al., 2005), and AD (McGeer et al., 2000). Researchers have observed upregulation of the complement system in human brain during AD and other neurodegenerative diseases (McGeer and McGeer, 1995; Yasojima et al., 1999). The complement system consists of distinct plasma proteins that act as opsonins and initiate a series of inflammatory responses (Janeway et al., 2001). Chemokines promote neuroinflammation by attracting leucocytes to the point of inflammation (Proost et al., 1996; Mélik-Parsadaniantz and Rostène, 2008). Neuroinflammation in turn has been implicated for the impairment of brain function (Campbell, 2004; Ownby, 2010; Tansey and Goldberg, 2010).

Further to the role of inflammasomes and humoral immune factors, several cellular immune factors such as granulocytes, monocytes/macrophages, NK cells, and T lymphocytes have also been shown to have a role in the pathophysiology of neuroinflammation (Petersen and Pedersen, 2005, 2006). The role of NK cells in various brain disorders such as depression, AD and PD has been reviewed and validated by some researchers (Poli et al., 2013). Likewise, the exchange of B cells across the BBB has been reported in patients with multiple sclerosis and associated with the development of autoimmunity in the CNS (von Büdingen et al., 2012).

## Discussion

### Neuroinflammation and cytokines

Neuroinflammation is an innate mechanism to ward off any stimuli that may be harmful to the host and has been shown to be mediated by various immune factors, particularly cytokines (Cacquevel et al., 2004) and chemokines (Ubogu et al., 2006). In particular, pro-inflammatory cytokines, such as TNF-α and the IL-1 family of cytokines, are credited for initiating the inflammatory reactions in the brain in response to an adverse stimulus, for continuation of neuroinflammation by attracting leucocytes at the site of inflammation and activating other pro-inflammatory factors, as well as for the anti-inflammatory pathway by enhancing IL-6 production that in turn stimulates production and expression of anti-inflammatory cytokines and immune factors (Cannon, 2000). However, what is more important here is the mechanism for the activation of these pro-inflammatory cytokines in response to an adverse stimulus in the first instance. While the concept of an increase in concentration of pro-inflammatory cytokines within the brain during aging and infection is now established, less is known about the mechanisms of cell signaling that result in the pro-inflammatory cytokine gradients within the brain.

### NLRP inflammasomes in neuroinflammation

While several theories have been postulated to explain this mechanism, recent findings suggest the role of inflammasomes to be important in particular for the IL-1 family of cytokines (Martinon and Tschopp, 2006; Stutz et al., 2009; Schroder and Tschopp, 2010). In response to PAMPs and DAMPs, trans-membranous TLRs that are present in semantic cells such as macrophages and dendritic cells, interact with NLRs on inflammasomes to recognize the stimulus, initiating an inflammasome cascade leading to the release of caspase 1 enzyme in the cytoplasm. Caspase 1 cleaves the pro-forms of the IL-1 family of cytokines to form their active forms (Inohara et al., 2005; Kanneganti et al., 2007; Franchi et al., 2009) that may result in neuroinflammation as a result of increased pro-inflammatory cytokine gradients. Although the acute neuroinflammatory response includes activation of resident tissue macrophages in the CNS and subsequent release of various cytokines and chemokines, this may also cause oxidative and nitrosative stress, which is a first line preventative mechanism against pathogenic extrinsic and intrinsic proteins and is less likely to cause long term damage to neurons (Frank-Cannon et al., 2009). However, it could still result in neurodegenerative changes, as well as in short-term cognitive impairment and exacerbated sickness behavior, as seen in rodent trials after LPS-induced acute neuroinflammation characterized by heightened pro-inflammatory cytokine response (Morimoto et al., 2002; Huang et al., 2008). Nevertheless, a bigger danger is posed by chronic neuroinflammation that is generally seen during old age (Sparkman and Johnson, 2008) and responsible for some brain pathologies such as depression (Wager-Smith and Markou, 2011), AD (Hauss-Wegrzyniak et al., 1998), PD (Tansey and Goldberg, 2010), and multiple sclerosis (Frischer et al., 2009).

For a chronic neuroinflammation to be sustained, the stimuli need to be continuous, potent and self-replicating. This could be explained from the findings that AD patients in old age suffer from a persistent degenerative condition that involves consistent increases in the various proposed etiologies, be it Aβ oligomerization or Tau phosphorylation, in the presence of neuroinflammation (Meraz-Ríos et al., 2013). A similar scenario could be plausible in the case of PD where formation of α-synuclein fibril aggregate increases in the presence of neuroinflammation. Accelerated formation of the mutant α-synuclein fibrils has been linked with the onset of PD (Conway et al., 1998, 2000). Although it has been shown that aggregated α-synuclein in microglia-like cells potentially activate the assembly of NLRP3 inflammasomes by inducing vesicle rupture in THP-1 cells that are sensed as danger signals (Cedillos, 2013), the opposite scenario still need to be studied (Cedillos, 2013). Consistent findings have also established the link between various etiologies of depression and neuroinflammation (Maes et al., 1997; Howren et al., 2009; Dowlati et al., 2010; Hannestad et al., 2011).

### A short note on the role of inflammasome mediated neuroinflammatory pathways in the comorbidity of systemic illnesses and psychiatric disorders

**Figure 4:**
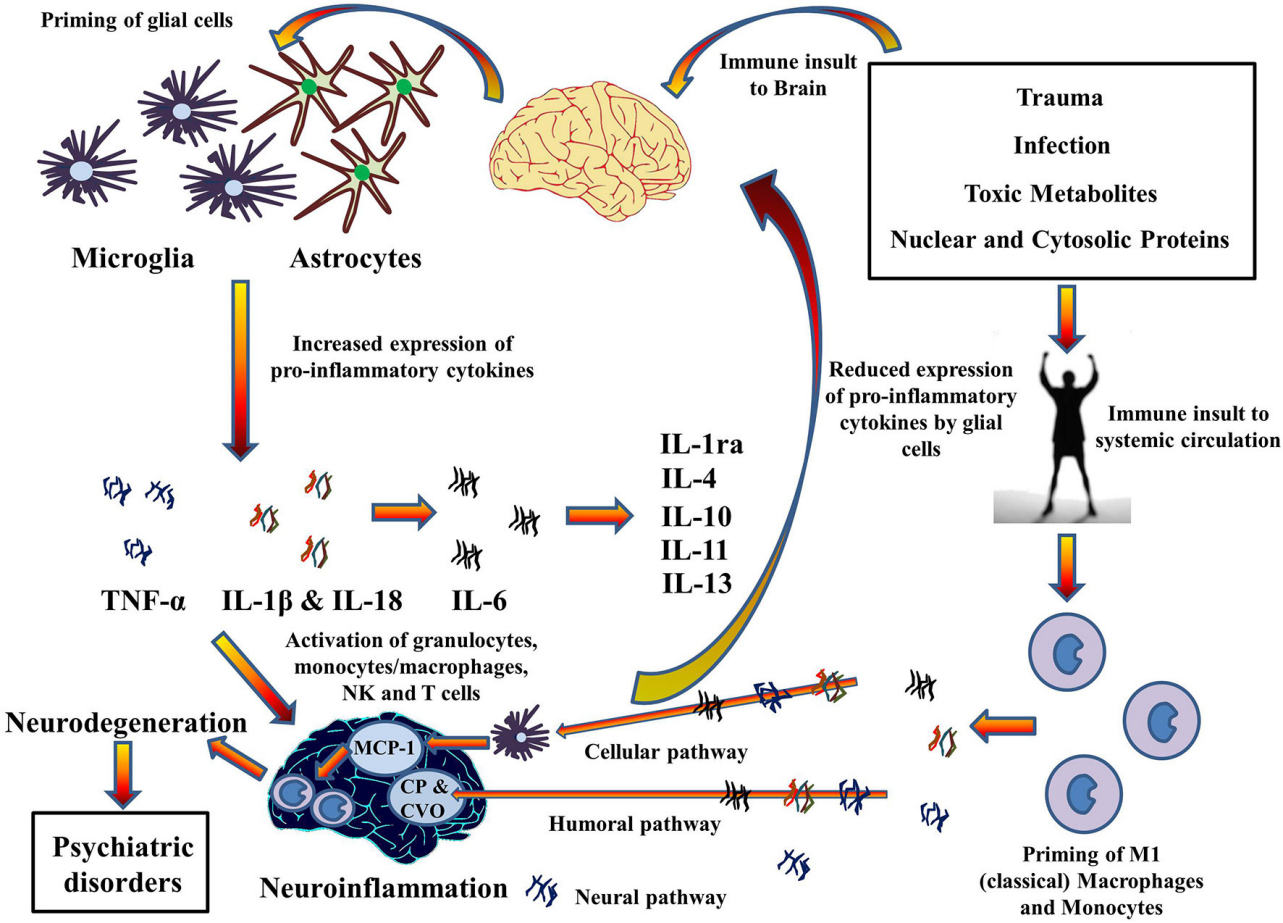
**Cytokines hypothesis of neuroinflammation: Implications in comorbidity of systemic illnesses with psychiatric disorders**. Pro-inflammatory cytokines can migrate between systemic circulation and brain in both directions which could explain the comorbidity of systemic illnesses with psychiatric disorders. There are three pathways for the transport of pro-inflammatory cytokines from systemic circulation to brain as described by Capuron and Miller (2011): Cellular, Humoral, and Neural. Moreover, PAMPs and DAMPs from trauma, infection, and metabolic waste can prime glial cells to express pro-inflammatory cytokines TNF-α, IL-1β, and IL-6. When expressed, these cytokines activates granulocytes, monocytes/macrophages, Natural Killer, and T cells and together contribute to the pathophysiology of neuroinflammation. Chronic neuroinflammation could result in neurodegeneration and associated psychiatric disorders. These pro-inflammatory cytokines also stimulate production and expression of anti-inflammatory cytokine by glial cells that function as negative feedback to reduce the expression of pro-inflammatory cytokines, subsiding the neuroinflammation. MCP-1, Monocyte chemoattractant protein-1; CP, Choroid plexus; CVO, Circumventricular organ.

High incidences of chronic inflammatory diseases such as cancer (Il'yasova et al., 2005), diabetes (De Rekeneire et al., 2006), osteoarthritis (Stannus et al., 2013), and cardiovascular disease (Volpato et al., 2001) have been demonstrated by prospective and correlative studies in aged cohorts. Investigation at the molecular level suggests increased levels of systemic pro-inflammatory cytokine IL-1β, in addition to TNF-α and IL-6, and acute phase proteins (e.g., CRP). Moreover, significant findings have confirmed an association between age related depression and level of pro-inflammatory cytokines in the brain (Godbout et al., 2008). This suggests a mechanism whereby pro-inflammatory cytokines migrate from systemic circulation to the brain and vice versa, especially during old age. Indeed, the pathways for the transport of pro-inflammatory cytokines to brain from systemic circulation have been described in a review by Capuron and Miller (2011) (See Figure 4). Rodent studies have shown increased production and expression of IL-1β in the brain after LPS-induced systemic inflammation (Cunningham et al., 2005) and changes in mood and behavior similar to depression after systemic administration of pro-inflammatory cytokines (Pollak and Yirmiya, 2002). This transport of pro-inflammatory cytokines into the brain and increase in their expression could be the reason for the comorbidity of systemic illnesses with psychiatric disorders in old age. Comorbid conditions, such as Type II diabetes (Grant and Dixit, 2013; Lee et al., 2013), obesity (Stienstra et al., 2011), cardiovascular diseases (Connat, 2011), and cancer (Fallowfield et al., 2001) with psychiatric illnesses therefore supports the hypothesis that inflammasomes play a large role in immunosenescence associated with aging and formation of psychiatric and systemic illnesses with age, the top-most reasons for deaths worldwide as mentioned by World Health Organization (2014). However, future research into the role of inflammasomes in these pathways during aging could possibly explain the link between age-related psychiatric and systemic illnesses.

The above mentioned link between systemic inflammatory conditions and CNS neurological disorders via activation of inflammasomes provides a molecular platform on which to develop therapies to prevent the initiation of those pro-inflammatory chronic cascades which are detrimental to the CNS. However, although hypothesized a number of times, it is yet to be seen if these therapies can be used to treat diseases such as cancer, diabetes, CVD, and auto-inflammatory disorders (Wilson and Cassel, 2010) that are major killers worldwide and comorbid with brain disorders. Inflammasomes, a molecular platform, could therefore be regarded as an advent to the innovation of therapies in the near future.

## Concluding remarks

Taken together, it is clear that the discovery of the role of inflammasomes in neuroinflammation has opened an array of research opportunities to investigate the inflammasome targeted therapies for age-related and pathological changes in the brain. It is also clear from the above discussion that the inflammasome activation pathway is complex and may involve the role of other immune factors such as cytokines, as well as mutant protein aggregates such as Aβ and α-synuclein fibrils. However, further research into these mechanisms and inflammasome-targeted therapies is advisable for constructing and assessing the complete profile of inflammasome-driven inflammatory pathways in brain.

### Conflict of interest statement

The presented work is supported by the National Health and Medical Research Council Australia (APP 1043771 to Bernhard T. Baune). The funders had no role in study design, data collection and analysis, decision to publish, or preparation of the manuscript. The authors declare that the research was conducted in the absence of any commercial or financial relationships that could be construed as a potential conflict of interest.
